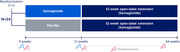# Investigating the effects of semaglutide in early Alzheimer’s disease using single‐cell transcriptomics and proteomics – rationale and design

**DOI:** 10.1002/alz.090259

**Published:** 2025-01-09

**Authors:** Peter Johannsen, Marie Bentsen, Kate Attfield, Kristian Steen Frederiksen, Giovanni B Frisoni, Rose Jeppesen, Ibrahim J Malik, Lotte Bjerre Knudsen

**Affiliations:** ^1^ Novo Nordisk A/S, Søborg, 2860 Søborg Denmark; ^2^ Novo Nordisk A/S, Søborg Denmark; ^3^ Oxford Centre for Neuroinflammation, Nuffield Department of Clinical Neurosciences, John Radcliffe Hospital, University of Oxford, Oxford, UK;, Oxford, Oxfordshire United Kingdom; ^4^ Danish Dementia Research Centre, Dept. of Neurology, Copenhagen University Hospital ‐ Rigshospitalet, Copenhagen Denmark; ^5^ Department of Clinical Medicine, Faculty of Health and Medical Sciences, University of Copenhagen, Copenhagen Denmark; ^6^ Laboratory of Neuroimaging of Aging (LANVIE), University of Geneva, Geneva Switzerland

## Abstract

**Background:**

Evidence suggests glucagon‐like peptide 1 receptor agonists (GLP‐1RAs) may have therapeutic potential in Alzheimer’s disease (AD). Cumulative evidence has indicated a potential reduction in cognitive decline in people with AD, while real‐world evidence has shown decreased dementia risk in patients with type 2 diabetes. Non‐clinical data reveal that GLP‐1RAs impact neuroinflammation and other biological processes believed to be involved in AD pathophysiology, including effects on central and peripheral immune cells. The safety and efficacy of the GLP‐1RA semaglutide in early AD (Clinical Dementia Rating global score 0.5–1) is being investigated in two phase 3 trials (evoke [NCT04777396] and evoke+ [NCT04777409]). The aim of this exploratory study is to investigate the effect of semaglutide on central (cerebrospinal fluid [CSF]) and peripheral (blood) inflammation in a similar population using single cell transcriptomics (i.e., assessment of gene expression in individual cells) to complement and expand on the molecular understanding of semaglutide and AD.

**Method:**

An interventional, randomized, parallel‐group, double‐blind, placebo‐controlled, multicenter, multinational study is designed to evaluate the effects of semaglutide versus placebo on central and peripheral inflammation in participants with early AD (NCT05891496; Figure). The 24 participants will be aged 55‐75 years and have established amyloid positivity. Central and peripheral inflammation will be investigated using single‐cell RNA sequencing, T‐cell receptor sequencing, and proteomics; AD‐related biomarkers (including glial fibrillary acidic protein and phosphorylated tau 217) will link cellular changes to AD pathology proxies. Cellular, protein, and biomarker changes will be assessed after 12 weeks of treatment.

**Result:**

The study is expected to read out at the end of 2024.

**Conclusion:**

Investigation of CSF and peripheral blood mononuclear cells at the single‐cell level in combination with protein and biomarker changes in the same individuals with early AD is an unprecedented opportunity to understand both the effects of semaglutide and the role of immune processes in early AD.